# Dental Anæsthesia by Cataphoresis

**Published:** 1896-03

**Authors:** William H. Rollins

**Affiliations:** Boston, Mass.


### DENTAL ANÆSTHESIA BY CATAPHORESIS.

BY WILLIAM H. ROLLINS, BOSTON, MASS.

Several articles have recently appeared in the dental journals on dental anæsthesia by cataphoresis. The impression which they convey is that this is a new thing. Now, on the contrary, it is at least thirty years old, for in 1859, Richardson extracted teeth under local anæsthesia produced by driving in medicines by the electric current. Even the use of cocaine, which is specially named in these articles, is old. Any one curious about this matter may turn to my report on dentistry in the Boston *Medical and Surgical Journal* for 1889, where he will find that McGraw used cocaine to produce anæsthesia of the dentine by cataphoresis. In fact, it will be hard at this late date to suggest any new applications of electricity in dental matters, the ground having been gone over too thoroughly.

Even bleaching teeth by electricity, which a recent writer claimed as new, has been in print since 1888. See my report on Dentistry in Boston *Medical and Surgical Journal* for that year, where it is credited to Ames. The treatment of alveolar abscess by electricity is seventeen years old, yet in the latest work on electro-therapeutics, the "International System of Electro-Therapeutics," edited by Bigelow, alveolar abscess is particularly mentioned as not a suitable subject for treatment by electricity. I expect to see the treatment of Riggs's disease and erosion by electricity rediscovered soon, as these methods have had about the usual time which I find it takes new dental ideas to diffuse themselves through the air with sufficient intensity to bring about this result.

As there is no doubt that the time is arriving when dental anæsthesia by cataphoresis will be more used than it has been, I wish to make some suggestions which are the result of long use of this agent. First, I have found by actual experience that the ordinary methods of using the street current are open to serious objections. Two dangers are always present,—risk of a short circuit and risk from return current through the ground.

The ordinary method, which physicians employ in electro-therapeutics, for reducing the current is a compact resistance on one wire. Turn to any recent work in which resistances are figured, and see how near the binding posts are to each other and how easy it is to cut out the resistance entirely by an instrument falling in contact with them, thus exposing the patient or the operator or both to the full current, which, to say the least, is very unpleasant. Or suppose the resistance is on the neutral main, and as these mains look alike and have no labels on them this may happen, then if the operator touch any one of the several terminals of other circuits which are needed about the modern dental chair, or even if he touch a gas burner, he or his patient or both may get a severe shock. When I first began to use the street current with the ordinary form of resistance I had this experience. To avoid all these and other dangers I arrange my resistance in another way, and do not use a single resistance or a compact resistance which can be easily short circuited. On each main I place thirty lamps, each of eight candle-power and in series. These two resistances are placed far apart at the top of the room where there is no possibility of a short circuit. This arrangement I call the minimum multiple resistance. On a hundred and ten volt circuit the maximum current that can pass at short circuit is four milliampères, which is the greatest current ever needed in dental electro-therapeutics. I also have a rotary resistance of a binary form, both sides being exactly alike and each side connected with one main. This further resistance, which I call the wave-maker, reduces the current to one one-hundredth of a milliampère, but by rotating it the full current which the minimum multiple resistance can transmit may be obtained gradually, or by stopping short of half a revolution any intermediate current may be obtained. With the resistance fixed in this way it is impossible for either the patient or the operator to get a current of greater strength than twice that which he is using, a circumstance of no moment. By rotating this wave-maker by means of a small electric motor I obtain the wave-current for treating the severe cramps of the muscles of the jaws which make long dental operations so painful to some patients. I do not know who first employed the wave-current in electro-therapeutics, but Kellogg has used it so long that it must be considered old. The point which I wish to make in this paper is that the periodical literature ought to be more carefully read, for as it is at present every original man, unless he is a really great man, must see his ideas buried for years (unless he patents them), and then credited to some one else. This is always a little discouraging, and deprives the profession of many useful things for a number of years.

